# Supplementary material for: Per- and Poly-Fluoroalkyl Substances in Portuguese Rivers: Spatial-Temporal Monitoring
Source: Molecules. 2023 Jan 26;28(3):1209. doi: 10.3390/molecules28031209 (PMC9921101; doi:10.3390/molecules28031209)
Supplement: Supplementary file 1 [file molecules-28-01209-s001.zip › molecules-2140262-supplementary_For conversion_edit(1) (1).pdf]

SUPPLEMENTARY MATERIAL

Per- and Poly-Fluoroalkyl Substances in Portuguese Rivers: Spatial-Temporal Monitoring

Marta O. Barbosa [1,2,3], Nuno Ratola [2,4], Vera Homem [2,4], M. Fernando R. Pereira [1,2], Adrián M. T. Silva [1,2], Ana R. L. Ribeiro [1,2], Marta Llorca [5,*] and Marinella Farré [5,*]

[1] LSRE-LCM—Laboratory of Separation and Reaction Engineering-Laboratory of Catalysis and Materials, Faculty of Engineering, University of Porto, Rua Dr. Roberto Frias, 4200-465 Porto, Portugal
[2] ALiCE—Associate Laboratory in Chemical Engineering, Faculty of Engineering, University of Porto, Rua Dr. Roberto Frias, 4200-465 Porto, Portugal
[3] Centre for Research and Intervention in Education (CIIE), Faculdade de Psicologia e de Ciências da Educação, Universidade do Porto, Rua Alfredo Allen s/n, 4200-135 Porto, Portugal
[4] LEPABE—Laboratory for Process Engineering, Environment, Biotechnology and Energy, Faculty of Engineering, University of Porto, Rua Dr. Roberto Frias, 4200-465 Porto, Portugal
[5] ON-HEALTH Research Group, Institute of Environmental Assessment and Water Research (IDAEA-CSIC), C/Jordi Girona, 18-26, 08034 Barcelona, Spain
* Correspondence: ML mlcqam@cid.csic.es (M.L.); mfuqam@cid.csic.es (M.F.)

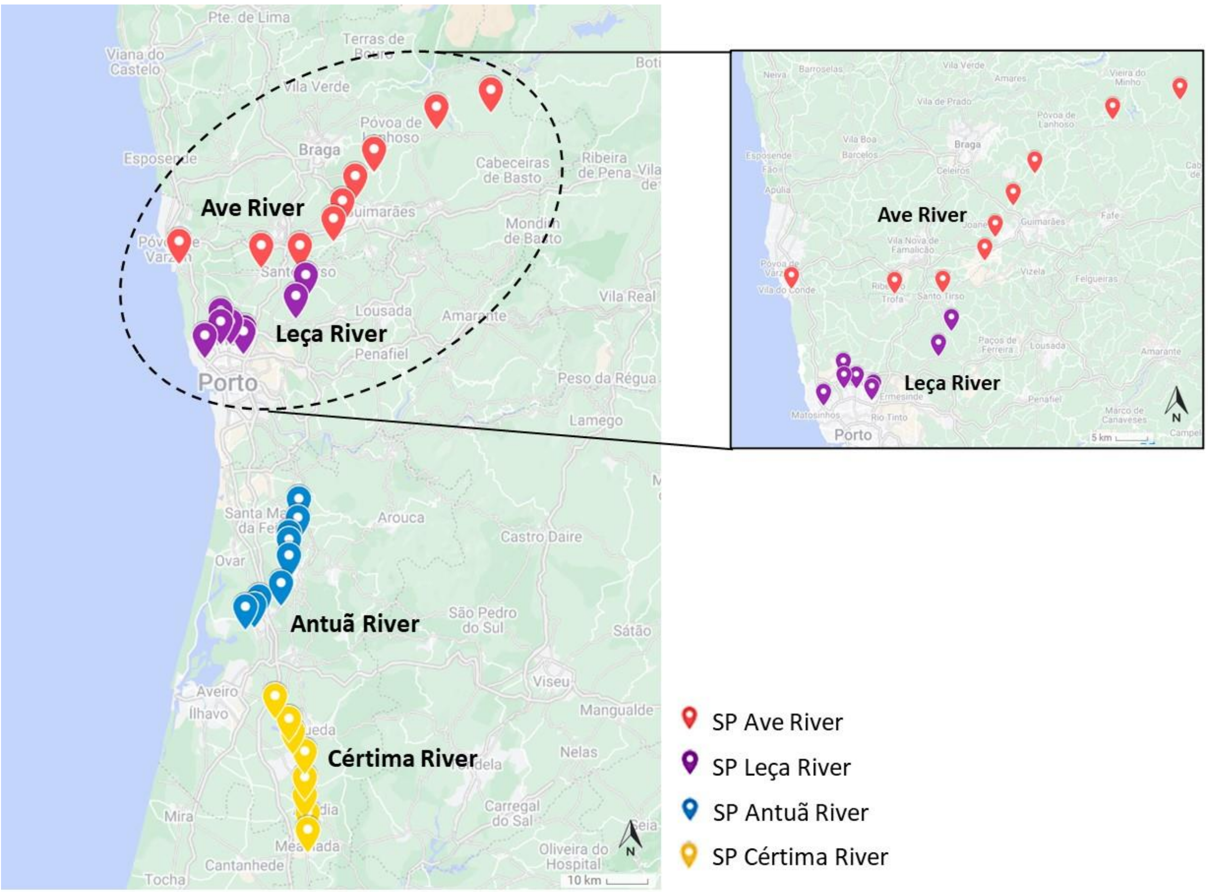

Figure S1. Target Portuguese rivers (Ave, Leça, Antuã, and Cértima) and location of sampling sites.

Table S1. Concentration (ng L^{-1}) of PFASs at different sampling points (SP) of the target Portuguese rivers, in the dry and wet seasons.

		Concentration (ng L^{-1})																	
		**PFBA**		**PFHpA**		**PFOA**		**PFNA**		**PFDA**		**PFTeA**		**PFHxS**		**PFOS**		**PFBS**	
**River**	**SP**	**Dry**	**Wet**	**Dry**	**Wet**	**Dry**	**Wet**	**Dry**	**Wet**	**Dry**	**Wet**	**Dry**	**Wet**	**Dry**	**Wet**	**Dry**	**Wet**	**Dry**	**Wet**
**Ave**	**1**	3.50	< MLOQ	< MLOD	< MLOQ	5.70	< MLOQ	0.60	< MLOD	< MLOD	< MLOD	< MLOD	< MLOD	< MLOD	< MLOD	< MLOD	< MLOD	0.20	< MLOQ
	**2**	< MLOD	< MLOQ	< MLOD	< MLOQ	5.45	0.94	< MLOD	< MLOD	< MLOD	< MLOD	< MLOD	< MLOD	< MLOD	< MLOD	< MLOD	< MLOD	< MLOD	< MLOQ
	**3**	< MLOD	< MLOQ	< MLOD	< MLOQ	4.45	0.51	< MLOD	< MLOD	< MLOD	< MLOD	< MLOD	< MLOD	< MLOD	< MLOD	< MLOD	< MLOD	< MLOD	< MLOQ
	**4**	< MLOD	< MLOQ	< MLOQ	< MLOQ	4.05	0.37	< MLOD	< MLOD	0.10	< MLOD	< MLOD	< MLOD	< MLOD	< MLOD	< MLOD	< MLOD	< MLOD	0.37
	**5**	< MLOD	< MLOQ	< MLOQ	< MLOQ	5.50	0.39	< MLOQ	< MLOD	1.80	< MLOD	< MLOD	< MLOD	< MLOD	< MLOD	< MLOD	< MLOQ	< MLOD	0.37
	**6**	< MLOD	< MLOQ	7.65	< MLOQ	7.95	1.88	< MLOQ	< MLOD	< MLOQ	< MLOD	< MLOD	< MLOD	< MLOD	< MLOD	0.80	< MLOQ	< MLOD	0.52
	**7**	< MLOD	< MLOQ	13.15	< MLOQ	14.50	4.13	2.30	< MLOQ	< MLOQ	< MLOQ	< MLOD	< MLOD	< MLOD	< MLOD	1.00	< MLOD	< MLOD	0.91
	**8**	< MLOD	< MLOQ	18.30	< MLOQ	15.15	2.87	2.55	< MLOQ	3.40	< MLOQ	< MLOD	< MLOD	< MLOD	< MLOD	1.55	< MLOQ	< MLOD	0.86
	**9**	< MLOD	< MLOQ	18.40	< MLOQ	12.85	2.22	< MLOQ	< MLOD	2.00	< MLOD	< MLOD	< MLOD	< MLOQ	< MLOD	< MLOQ	< MLOQ	< MLOQ	1.12
**Leça**	**1**	< MLOD	< MLOQ	< MLOD	< MLOQ	1.65	0.08	< MLOD	< MLOQ	< MLOD	< MLOQ	< MLOD	< MLOD	< MLOD	< MLOD	< MLOD	< MLOD	< MLOD	< MLOD
	**2**	< MLOD	< MLOQ	< MLOQ	< MLOQ	2.95	10.35	< MLOD	< MLOQ	< MLOD	< MLOD	< MLOD	< MLOD	< MLOD	< MLOD	< MLOD	< MLOD	< MLOD	< MLOD
	**3**	< MLOD	< MLOQ	< MLOD	< MLOQ	11.45	1.81	2.10	< MLOQ	1.90	< MLOQ	< MLOD	< MLOD	< MLOQ	< MLOD	4.45	1.89	< MLOQ	0.56
	**4**	< MLOD	< MLOQ	< MLOD	< MLOQ	< MLOQ	1.78	< MLOD	< MLOQ	< MLOD	< MLOQ	< MLOD	< MLOD	< MLOQ	< MLOD	< MLOQ	2.01	< MLOQ	0.74
	**5**	0.20	< MLOQ	< MLOD	< MLOQ	< MLOQ	4.14	< MLOD	< MLOQ	< MLOQ	< MLOQ	< MLOD	< MLOD	< MLOQ	< MLOD	4.95	3.50	< MLOQ	0.71
	**6**	< MLOD	< MLOQ	< MLOD	< MLOQ	< MLOQ	1.28	< MLOD	< MLOQ	< MLOQ	< MLOQ	< MLOD	< MLOD	< MLOQ	0.31	8.90	3.49	< MLOQ	0.95
	**7**	< MLOD	< MLOQ	< MLOD	< MLOQ	< MLOQ	1.78	< MLOQ	< MLOQ	2.75	< MLOQ	4.85	< MLOD	< MLOQ	0.39	6.65	4.47	< MLOQ	1.33
	**8**	< MLOD	< MLOQ	< MLOD	< MLOQ	14.50	5.38	< MLOQ	< MLOQ	< MLOQ	< MLOQ	< MLOD	< MLOD	< MLOQ	0.35	5.85	5.38	< MLOQ	1.34
**Antuã**	**1**	< MLOD	22.57	< MLOQ	< MLOQ	3.35	1.63	< MLOD	< MLOD	< MLOQ	< MLOD	< MLOD	< MLOD	< MLOD	< MLOD	< MLOD	< MLOQ	0.70	< MLOQ
	**2**	< MLOD	5.91	< MLOD	< MLOQ	3.80	1.67	< MLOD	< MLOD	0.20	< MLOD	< MLOD	< MLOD	< MLOD	< MLOD	< MLOQ	< MLOQ	< MLOD	0.53
	**3**	< MLOD	5.89	< MLOD	< MLOQ	16.95	8.97	< MLOQ	< MLOD	< MLOQ	< MLOD	5.45	< MLOD	< MLOD	< MLOD	1.70	< MLOQ	< MLOD	0.63
	**4**	< MLOD	< MLOQ	< MLOD	< MLOQ	14.70	7.76	< MLOD	< MLOD	< MLOQ	< MLOD	< MLOD	< MLOD	< MLOD	< MLOD	< MLOQ	4.61	< MLOD	0.60

	**5**	< MLOD	< MLOQ	< MLOD	< MLOQ	14.05	6.18	< MLOD	< MLOQ	< MLOQ	< MLOQ	< MLOD	< MLOD	< MLOQ	< MLOD	3.45	3.93	< MLOQ	0.43
	**6**	< MLOD	< MLOQ	< MLOD	< MLOQ	7.85	4.99	< MLOD	< MLOQ	< MLOD	< MLOQ	< MLOD	< MLOD	< MLOD	< MLOD	< MLOQ	2.79	< MLOD	< MLOQ
	**7**	< MLOD	< MLOQ	< MLOD	< MLOQ	11.75	5.49	1.00	< MLOQ	< MLOQ	< MLOQ	< MLOD	< MLOD	< MLOQ	< MLOD	< MLOQ	< MLOQ	< MLOQ	0.46
	**8**	< MLOD	< MLOQ	< MLOD	< MLOQ	14.25	2.31	0.85	< MLOQ	< MLOQ	< MLOQ	< MLOD	< MLOD	< MLOQ	< MLOD	1.70	1.79	< MLOQ	0.41
	**9**	< MLOD	< MLOQ	< MLOD	< MLOQ	9.60	4.74	< MLOQ	< MLOQ	0.25	< MLOQ	< MLOD	< MLOD	< MLOQ	< MLOD	< MLOQ	2.43	< MLOQ	0.39
**Cértima**	**1**	< MLOD	4.00	< MLOD	< MLOQ	< MLOQ	0.67	< MLOQ	< MLOD	< MLOD	< MLOD	< MLOD	< MLOD	< MLOQ	< MLOD	< MLOQ	< MLOD	< MLOQ	1.54
	**2**	< MLOD	9.20	< MLOD	< MLOQ	< MLOQ	0.59	< MLOD	< MLOQ	< MLOQ	< MLOQ	< MLOD	< MLOD	< MLOQ	< MLOD	< MLOD	< MLOQ	< MLOQ	0.70
	**3**	1.20	3.93	< MLOD	< MLOQ	5.25	1.13	< MLOQ	< MLOD	< MLOQ	< MLOD	< MLOD	< MLOD	< MLOQ	< MLOD	< MLOD	< MLOQ	< MLOQ	0.66
	**4**	3.30	5.81	< MLOD	< MLOQ	3.85	0.23	1.85	< MLOD	1.05	< MLOD	< MLOD	< MLOD	< MLOD	< MLOD	< MLOD	< MLOQ	< MLOD	0.40
	**5**	4.60	3.98	< MLOD	< MLOQ	10.05	1.05	2.05	< MLOD	1.75	< MLOD	< MLOD	< MLOD	< MLOD	< MLOD	< MLOQ	< MLOQ	< MLOD	< MLOQ
	**6**	8.50	5.15	< MLOD	< MLOQ	4.70	1.96	0.90	< MLOD	3.35	< MLOD	< MLOD	< MLOD	1.45	< MLOD	22.65	6.39	6.90	0.99
	**7**	4.65	8.44	5.10	< MLOQ	8.05	2.62	1.80	< MLOD	1.10	< MLOD	< MLOD	< MLOD	0.70	< MLOD	11.80	7.44	6.10	0.95
	**8**	3.70	5.64	13.70	< MLOQ	5.30	3.85	1.85	< MLOQ	0.55	< MLOQ	< MLOD	< MLOD	0.50	< MLOD	6.35	6.48	7.05	1.05

MLOD - method limit of detection; MLOQ - method limit of quantification.

Table S2. Extended survey of the occurrence data for the targeted PFASs in European surface water samples (ng L^{-1}); nd, not detected.

Country	River	PFBA	PFPeA	PFHxA	PFHpA	PFOA	PFNA	PFDA	PFUnDA	PFDoDA	PFTrDA	PFTeDA	PFBS	PFHxS	PFOS	REF.
Portugal	Ave (present study)	0.20	nd	nd	3.32	4.94	0.36	0.41	nd	nd	nd	nd	0.24	nd	0.19	This study
Portugal	Leça (present study)	0.02	nd	nd	0.11	3.58	0.26	0.30	nd	nd	nd	0.3	0.36	0.07	3.22	This study
Portugal	Antuã (present study)	1.91	nd	nd	0.11	7.78	0.19	0.04	nd	nd	nd	0.3	0.23	nd	1.25	This study
Portugal	Cértima (present study)	4.51	nd	nd	1.28	3.08	0.56	0.50	nd	nd	nd	nd	0.23	nd	nd	This study
Austria	Danube	-	-	2.06	2	18	0	0	0	0	-	-	-	4.33	-	[1]
Austria	Schwechat	-	-	5.00	1.67	3.77	0.8	0.77	0	0	-	-	-	20.37	-	[1]
Austria	Liesing	-	-	1.97	2.63	10.33	1.3	1.2	0	0	-	-	-	15.33	-	[1]
France	Rhône	-	-	81.46	-	2.53	-	-	-	-	-	-	-	1.27	3.23	[2]
Germany	White Elster	0.5	0.25	1	-	13.9	1.25	5	0.5	-	-	-	1.25	0.5	1	[3]
Germany	Saale	0.5	1.1	3.8	1.1	21.2	3.8	2.2	3.3	-	-	-	-	-	2.2	[3]
Germany	Elbe	2.7	1.1	1.6	0.3	2.4	0.8	-	-	-	-	-	-	0.54	-	[3]
Germany	Rhine	4.31	2.58	2.94	1.22	2.63	0.42	0.37	0.06	0.03	0.04	0.01	21.9	1.98	13.8	[4]
Ireland	Nore	-	301	134	6.9	173	43	22	-	-	-	-	49.5	-	-	[5]
Ireland	Suir	-	424	173	5.5	205	46	24	-	-	-	-	68.9	-	-	[5]
Ireland	Liffey	-	250	123	6.9	138	31	16	-	-	-	-	64	-	-	[5]
Ireland	Annalee	-	269	133	8.8	197	56	24	-	-	-	-	35	-	-	[5]
Italy	Olona	8.1	6.6	11.46	15.02	79.5	26.95	24.1	11.9	1.9	-	-	17.4	3.6	16.9	[6]
Italy	Seveso	2.5	3.75	4.25	6.75	34.5	8.75	7	2.75	2	-	-	10	3.75	9.75	[6]
Italy	Lambro	4.88	2.59	2.08	3.68	20.82	7.25	7.68	2.38	1.5	-	-	5.47	4.29	14.32	[6]
Spain	Guadalquivir	214.3	8.6	-	4.3	11.6	5.1	1.0	-	-	-	nd	10.1	4.1	1.8	[7]

Spain	Jucar	49.87	0.38	3.82	2.22	4.36	2.31	14.21	0.04	nd	0	0.01	nd	3.25	11.29	[8]
Spain	Ebro	35.2	1.1	1.7	2.0	7.3	0.4	0.7	-	0.3	-	0.3	nd	0.5	2.2	[7]
Spain	Llobregat	19.5	0.40	2.43	6.16	20.3	3.87	0.41	0.01	nd	0.7	nd	0.32	6.7	234	[9]
Sweden	Torne älv	0.94	-	0.25	1.35	0.26	0.15	0.13	0.077	0.053	0.015	-	0.39	0.069	0.082	[10]
Sweden	Kalix älv	0.76	-	0.25	1.35	0.23	0.23	0.015	0.10	0.10	0.14	-	0.015	0.015	0.053	[10]
Sweden	Råne älv	1.2	-	0.25	1.35	0.21	1.3	0.28	0.16	0.19	0.15	-	0.015	0.15	0.34	[10]
Sweden	Lule älv	0.60	-	0.25	1.35	0.39	0.23	0.14	0.073	0.033	0.015	-	0.015	0.051	0.058	[10]
Sweden	Alterälven	0.60	-	0.25	0.61	0.043	0.18	0.11	0.044	0.015	0.015	-	0.015	0.015	0.12	[10]
Sweden	Pite älv	0.97	-	0.25	0.54	0.44	0.40	0.22	0.081	0.069	0.015	-	4.1	0.31	0.28	[10]
Sweden	Skellefte älv	0.91	-	0.25	1.35	0.39	0.36	0.27	0.17	0.12	0.015	-	5.1	0.19	0.30	[10]
Sweden	Rickleån	0.99	-	0.25	1.35	0	0.09	0.015	0.04	0.015	0.015	-	0.03	0.015	0.04	[10]
Sweden	Ume älv	0.63	-	0.25	1.35	0.26	0.32	0.064	0.058	0.015	0.015	-	0.048	0.14	0.25	[10]
Sweden	Ume älv [Gubböle]	0.57	-	0.25	1.0	3.3	5.8	4.4	1.6	0.77	0.46	-	16	18	6.9	[10]
Sweden	Vindelälven	0.47	-	0.25	1.35	0.043	0.26	0.015	0.074	0.067	0.094	-	0.015	0.015	0.074	[10]
Sweden	Vindelälven	0.99	-	0.25	1.35	0.043	0.091	0.015	0.041	0.015	0.015	-	0.030	0.015	0.042	[10]
Sweden	Öre älv	0.62	-	0.25	1.35	0.39	0.36	0.14	0.084	0.043	0.015	-	0.17	0.12	0.18	[10]
Sweden	Lögde älv	0.96	-	0.25	1.35	0.043	0.16	0.015	0.015	0.015	0.015	-	0.015	0.015	0.060	[10]
Sweden	Gide älv	0.79	-	0.25	1.35	0.043	0.12	0.015	0.015	0.015	0.015	-	0.015	0.015	0.015	[10]
Sweden	Ångermanälven	0.96	-	0.25	1.35	0.043	0.40	0.12	0.10	0.015	0.015	-	0.015	0.015	0.16	[10]
Sweden	Indalsälven	1.0	-	0.25	0.36	1.5	2.8	1.9	0.84	0.40	0.84	-	6.9	7.6	3.1	[10]
Sweden	Ljungan	1.2	-	0.25	1.35	0.043	0.21	0.024	0.044	0.015	0.015	-	0.015	0.015	0.12	[10]
Sweden	Delångersån	1.9	-	0.25	1.4	3.6	5.6	4.3	1.8	0.82	0.41	-	17	16	6.8	[10]
Sweden	Ljusnan	1.1	-	0.25	1.35	0.34	0.33	0.092	0.087	0.035	<0.02	-	0.44	0.10	0.046	[10]
Sweden	Gavleån	2.1	-	1.1	0.82	0.92	0.41	0.17	0.078	0.015	0.015	-	4.9	0.38	0.29	[10]
Sweden	Dalälven	1.2	-	1.1	0.56	0.40	0.37	0.15	0.16	0.046	0.42	-	0.42	0.13	0.61	[10]
Sweden	Fyrisån	2.5	-	4.2	1.4	4.2	0.63	0.34	0.092	0.034	0.015	-	2.2	12	5.3	[10]

Sweden	Norrström	2.9	-	1.9	1.3	1.6	0.51	0.22	0.075	0.061	0.015	-	0.83	1.2	2.4	[10]
Sweden	Nyköpingsån	3.7	-	1.5	1.4	1.1	0.44	0.18	0.093	0.015	0.093	-	0.61	0.64	0.71	[10]
Sweden	Motala ström	2.1	-	0.25	1.35	0.61	0.32	0	0.047	0.015	0.015	-	0.015	0.29	0.63	[10]
Sweden	Botorpsström	2.0	-	0.25	1.35	0.22	0.19	0.015	0.057	0.015	0.015	-	0.015	0.015	0.11	[10]
Sweden	Emån	2.3	-	0.51	1.7	3.8	5.4	3.7	1.5	0.70	1.5	-	12	14	5.1	[10]
Sweden	Alsterån	1.6	-	0.25	1.35	0.42	0.38	0.038	0.097	0.015	0.015	-	0.015	0.015	0.25	[10]
Sweden	Ljungbyån	1.5	-	1.3	0.74	0.91	0.19	0.065	0.051	0.015	0.015	-	0.042	0.46	0.28	[10]
Sweden	Lyckebyån	1.9	-	0.25	0.89	0.64	0.45	0.17	0.12	0.031	0.015	-	0.015	0.47	1.1	[10]
Sweden	Mörrumsån	1.8	-	0.25	0.85	0.98	0.60	0.22	0.14	0.080	0.015	-	0.51	0.42	0.60	[10]
Sweden	Helge Å	1.8	-	1.5	1.0	1.2	0.61	0.30	0.18	0.095	0.015	-	19	1.9	0.54	[10]
Sweden	Kävlingeån	1.8	-	2.6	0.96	1.1	0.12	0.088	0.037	0.015	0.015	-	0.15	0.73	0.50	[10]
Sweden	Rönneån	2.3	-	1.6	0.93	1.3	0.26	0.015	0.015	0.015	0.015	-	0.87	3.0	3.9	[10]
Sweden	Lagan	1.8	-	0.25	1.35	1.0	0.36	0.058	0.018	0.016	0.015	-	0.083	0.34	0.56	[10]
Sweden	Nissan	2.7	-	1.1	0.85	1.2	0.52	0.11	0.050	0.030	0.015	-	0.44	0.41	0.81	[10]
Sweden	Ätran	1.5	-	0.25	1.35	0.69	0.33	0.045	0.015	0.015	0.015	-	0.015	0.20	0.35	[10]
Sweden	Viskan	1.4	-	1.3	0.95	1.6	0.54	0.19	0.081	0.015	0.015	-	0.015	0.39	0.79	[10]
Sweden	Göta älv	1.3	-	0.25	0.55	1.1	0.41	0.11	0.039	0.015	0.015	-	0.041	0.34	0.79	[10]
The Netherlands	Waal	7,5	5.3	5.5	1.86	4.2	0.7	0.44	-	-	-	-	20.2	2	3.7	[11]
UK	Thames	6.96	15.7	12.2	4.1	8.51	1.18	0.86	0.07	0.04	-	-	5.06	7.14	13.8	[4]

Table S3. Target compounds, acronym, CAS number, structure, and relative molecular mass (M_r).

Compound	Acronym	CAS number	Structure
Perfluorobutanoic acid	PFBA	375-22-4	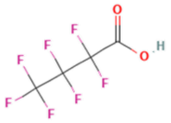
Perfluoropentanoic acid	PFPeA	2706-90-3	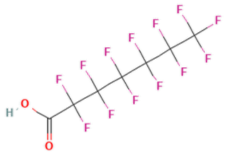
Perfluorohexanoic acid	PFHxA	307-24-4	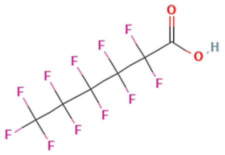
Perfluoroheptanoic acid	PFHpA	375-85-9	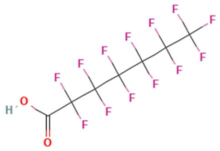
Perfluorooctanoic acid	PFOA	335-67-1	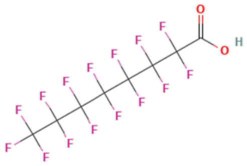
Perfluorononanoic acid	PFNA	375-95-1	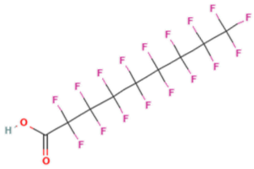
Perfluorodecanoic acid	PFDA	335-76-2	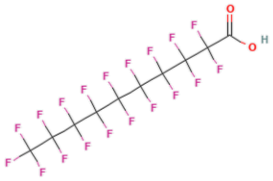
Perfluoroundecanoic acid	PFUnA	2058-94-8	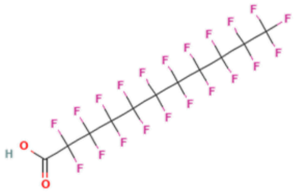
Perfluorododecanoic acid	PFDoDA	307-55-1	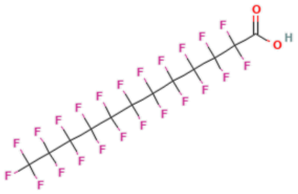

Perfluorotridecanoic acid	PFTrDA	72629-94-8	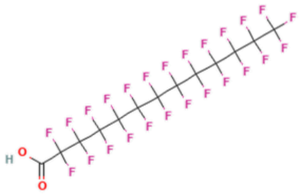
Perfluorotetradecanoic acid	PFTeDA	376-06-7	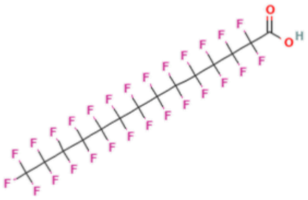
Perfluorohexadecanoic acid	PFHxDA	67905-19-5	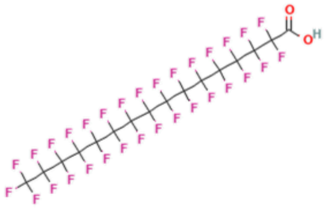
Perfluorooctadecanoic acid	PFODA	16517-11-6	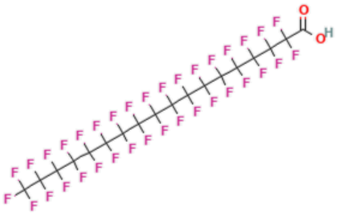
Perfluorobutanesulfonic acid	PFBS	375-73-5	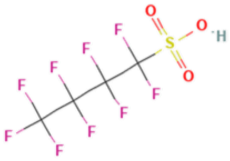
Perfluorohexasulfonic acid	PFHxS	355-46-4	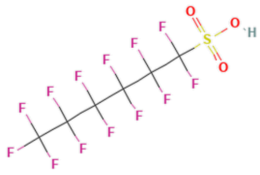
Perfluorooctanesulfonic acid	PFOS	1763-23-1	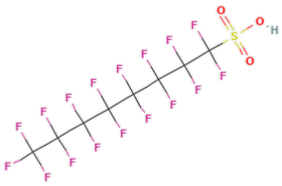
Perfluorodecanesulfonic acid	PFDS	335-77-3	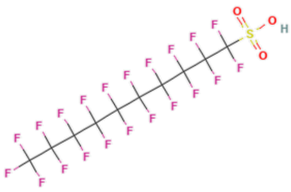

Perfluorooctanesulfonamide	PFOSA	754-91-6	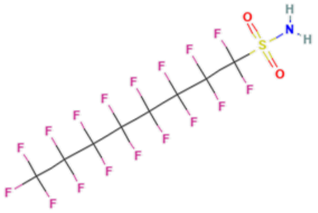

Table S4. Summary of accurate masses from selected PFASs and their product ions detected in the MS/HRMS spectra.

Analyte	m/z		
	Precursor ion	1st transition	2nd transition
**PFBA**	213	169	119
**PFPeA**	263	219	69
**PFHxA**	313	269	169
**PFHpA**	363	319	169
**PFOA**	413	369	169
**PFNA**	463	219	169
**PFDA**	513	469	169
**PFUdA**	563	519	169
**PFDoA**	613	569	169
**PFTeDA**	713	669	169
**PFHxDA**	813	769	169
**PFODA**	913	869	169
**PFBS**	299	80	99
**PFHxS**	399	80	99
**PFOS**	499	80	99
**PFDS**	599	80	99
**PFOSA**	498	78	498
**PFHxPA**	399	79	399
**PFOPA**	499	79	499
**PFDPA**	599	79	599

1st transition: quantification ion

2nd transition: confirmation ion

Table S5. Retention time, linearity and method limit of quantification (MLOQ) for each target analyte.

Analyte	Retention time (min)	Instrumental linearity range (µg L^{-1})	MLOQ (ng L^{-1})
PFBA	0.67	5 - 100	0.040
PFPeA	1.04	5 - 100	0.040
PFHxA	2.24	5 - 100	0.400
PFHpA	4.24	5 - 100	0.400
PFOA	5.52	0.5 - 100	0.040
PFNA	6.34	0.5 - 100	0.040
PFDA	6.97	0.5 - 100	0.040
PFUnDA	7.50	1 - 100	0.040
PFDoDA	7.95	0.5 - 100	0.040
PFTrDA	8.34	5 - 100	0.100
PFTeDA	8.71	5 - 100	0.100
PFHxDA	9.20	5 - 100	0.100
PFODA	10.15	20 - 100	0.800
PFBS	1.48	0.5 - 100	0.020
PFHxS	3.17	0.1 - 100	0.004
PFOS	6.45	0.1 - 100	0.004
PFDS	7.50	0.1 - 100	0.004
PFOSA	6.10	10 - 100	0.200

Table S6. Measured environmental concentration (MEC; mg L^{-1}), *i.e.*, the highest concentration of PFASs, found in the target Portuguese rivers and respective sampling point (SP) and season.

Compound	MEC (mg L^{-1})	River, SP and season
PFBA	2.26E-05	Antuã, SP1, Wet
PFHxS	1.45E-06	Cértima, SP6, Dry
PFBS	7.05E-06	Cértima, SP8, Dry
PFHpA	1.84E-05	Ave, SP9, Dry
PFOA	1.70E-05	Antuã, SP3, Dry
PFNA	2.55E-06	Ave, SP8, Dry
PFDA	3.40E-06	Ave, SP8, Dry
PFTeA	5.45E-06	Antuã, SP3, Dry
PFOS	2.27E-05	Cértima, SP6, Dry

References

[1] M. Clara, O. Gans, S. Weiss, D. Sanz-Escribano, S. Scharf, C. Scheffknecht, Perfluorinated alkylated substances in the aquatic environment: An Austrian case study, Water Research, 43 (2009) 4760-4768.

[2] N. Schmidt, V. Fauvelle, J. Castro-Jiménez, K. Lajaunie-Salla, C. Pinazo, C. Yohia, R. Sempéré, Occurrence of perfluoroalkyl substances in the Bay of Marseille (NW Mediterranean Sea) and the Rhône River, Marine Pollution Bulletin, 149 (2019) 110491.

[3] U. Shafique, S. Schulze, C. Slawik, A. Böhme, A. Paschke, G. Schüürmann, Perfluoroalkyl acids in aqueous samples from Germany and Kenya, Environ Sci Pollut Res Int, 24 (2017) 11031-11043.

[4] Y. Pan, H. Zhang, Q. Cui, N. Sheng, L.W.Y. Yeung, Y. Sun, Y. Guo, J. Dai, Worldwide Distribution of Novel Perfluoroether Carboxylic and Sulfonic Acids in Surface Water, Environmental Science & Technology, 52 (2018) 7621-7629.

[5] B. Huerta, B. McHugh, F. Regan, Development and application of an LC-MS method to the determination of poly- and perfluoroalkyl substances (PFASs) in drinking, sea and surface water samples, Analytical Methods, 14 (2022) 2090-2099.

[6] S. Castiglioni, S. Valsecchi, S. Polesello, M. Rusconi, M. Melis, M. Palmiotto, A. Manenti, E. Davoli, E. Zuccato, Sources and fate of perfluorinated compounds in the aqueous environment and in drinking water of a highly urbanized and industrialized area in Italy, Journal of Hazardous Materials, 282 (2015) 51-60.

[7] M. Lorenzo, J. Campo, M. Farré, F. Pérez, Y. Picó, D. Barceló, Perfluoroalkyl substances in the Ebro and Guadalquivir river basins (Spain), Science of the Total Environment, 540 (2016) 191-199.

[8] J. Campo, M. Lorenzo, F. Pérez, Y. Picó, M.l. Farré, D. Barceló, Analysis of the presence of perfluoroalkyl substances in water, sediment and biota of the Jucar River (E Spain). Sources, partitioning and relationships with water physical characteristics, Environmental Research, 147 (2016) 503-512.

[9] J. Campo, F. Pérez, A. Masiá, Y. Picó, M.l. Farré, D. Barceló, Perfluoroalkyl substance contamination of the Llobregat River ecosystem (Mediterranean area, NE Spain), Science of The Total Environment, 503-504 (2015) 48-57.

[10] M.A. Nguyen, K. Wiberg, E. Ribeli, S. Josefsson, M. Futter, J. Gustavsson, L. Ahrens, Spatial distribution and source tracing of per- and polyfluoroalkyl substances (PFASs) in surface water in Northern Europe, Environmental Pollution, 220 (2017) 1438-1446.

[11] W.A. Gebbink, L. van Asseldonk, S.P.J. van Leeuwen, Presence of Emerging Per- and Polyfluoroalkyl Substances (PFASs) in River and Drinking Water near a Fluorochemical Production Plant in the Netherlands, Environmental Science & Technology, 51 (2017) 11057-11065.
